# 652. Comparison of QuantiFERON-TB Gold Plus and T-SPOT.TB in the Diagnosis of Active Tuberculosis

**DOI:** 10.1093/ofid/ofab466.849

**Published:** 2021-12-04

**Authors:** Lifan Zhang, zhengrong yang, Xiaoqing Liu, Huimin Ma, Qiping Ge, Yueqiu Zhang, Xiaochun Shi, Qifei Cao, Mengqiu Gao

**Affiliations:** 1 Peking Union Medical College Hospital, Beijing, China; 2 Peking Union Medical College Hospital, Chinese Academy of Medical Sciences and Peking Union Medical College, Beijing, China; 3 Beijing Chest Hospital, Capital Medical University/Beijing, Beijing, China

## Abstract

**Background:**

**Objective:** To compare the accuracy of Quantiferon ®- TB Gold Plus (QFT-Plus) and T-SPOT.TB in the diagnosis of active tuberculosis (ATB).

**Methods:**

From April 2020 to May 2021, patients with pathologically confirmed or clinically diagnosed ATB in Peking Union Medical College Hospital and Beijing Chest Hospital were enrolled as case group, while patients excluded from ATB in the same period were enrolled as control group, The clinical and laboratory information were collected. QFT-Plus and T-SPOT. TB were tested simultaneously to evaluate the consistency of the results and compare the sensitivity, specificity, predictive values and likelihood ratios for diagnosing ATB.

**Results:**

Fifty-seven ATB patients and 159 non-ATB patients were included. 33 (57.9%) ATB patients were pathologically confirmed. The proportions of indeterminate results in QFT-Plus and T-SPOT.TB were 3.7% vs 0%, respectively. The agreement between the results of the QFT-Plus and T-SPOT.TB was substantial (kappa=0.644, p＜0.001). The area under the ROC curve of the QFT-Plus and T-SPOT.TB for diagnosing ATB was 0.759 (95%CI 0.689-0.829) vs 0.810 (95%CI 0.742-0.877), respectively, and there was no significant difference (p=0.303). the sensitivity of the QFT-Plus and T-SPOT.TB was 85.2% vs 89.5%, while the specificity was 61.7% vs 52.2%, respectively. In 33 Patients with pathologically confirmed ATB, the sensitivity of QFT-Plus and T-SPOT.TB was 87.9% vs 93.9% (p=0.669), respectively. In 78 patients (36.1%) who received glucocorticoid / immunosuppressive / biological agents, the positive rate of QFT-Plus was 35.9% (28/78), which was significantly lower than that of those who did not receive these agents (77 / 138pm,55.8%) (p=0.005), but there was no significant difference in the positive rate of T-SPOT.TB between the two groups (52.6% vs. 62.3%, p=0.162). The positive rate for both tests was independent of the peripheral blood lymphocyte count (p=0.675 for QFT-Plus vs. P=0.138 for T-SPOT.TB).

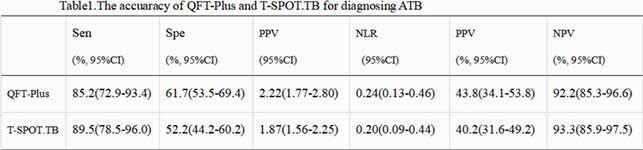

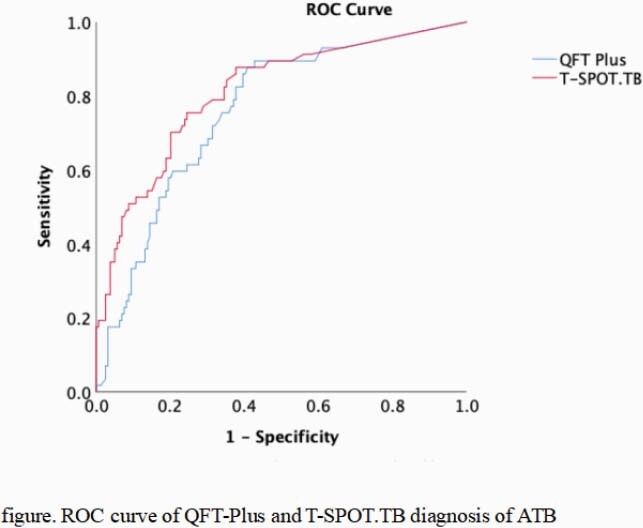

**Conclusion:**

There was no significant difference between the QFT-Plus and T-SPOT.TB in the diagnosis of ATB. QFT-plus might be prone to indeterminate results and influenced by the immunosuppressive status. The results need to be verified by a prospective cohort study with large sample.

**Disclosures:**

**All Authors**: No reported disclosures

